# Supplementary material for: Changes in the Mitochondrial Dynamics and Functions Together with the mRNA/miRNA Network in the Heart Tissue Contribute to Hypoxia Adaptation in Tibetan Sheep
Source: Animals (Basel). 2022 Feb 25;12(5):583. doi: 10.3390/ani12050583 (PMC8909807; doi:10.3390/ani12050583)
Supplement: Supplementary file 1 [file animals-12-00583-s001.zip › Supplementary material 1/Supplementary Figure.pdf]

Supplementary Figures:

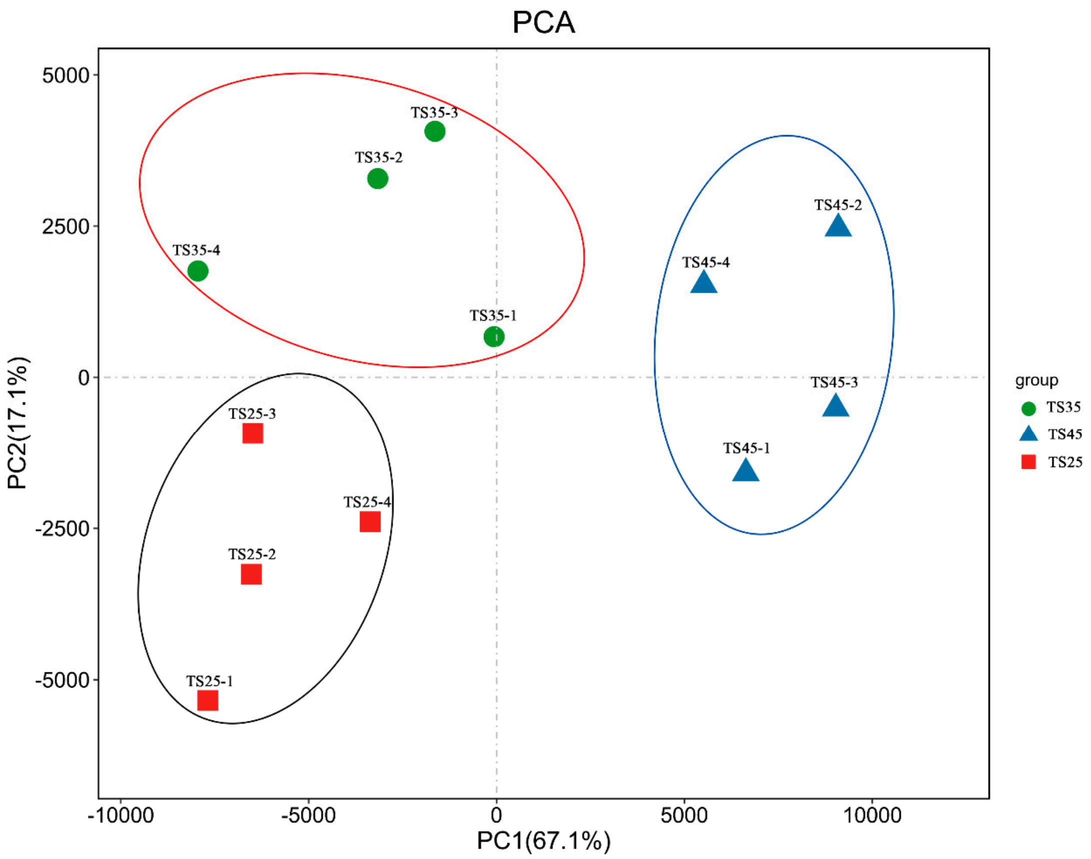

Figure S1. Principal component analysis results among samples. (**TS25**) 2,500 m altitude Tibetan sheep. (**TS35**) 3,500 m altitude Tibetan sheep. (**TS45**) 4,500 m altitude Tibetan sheep.

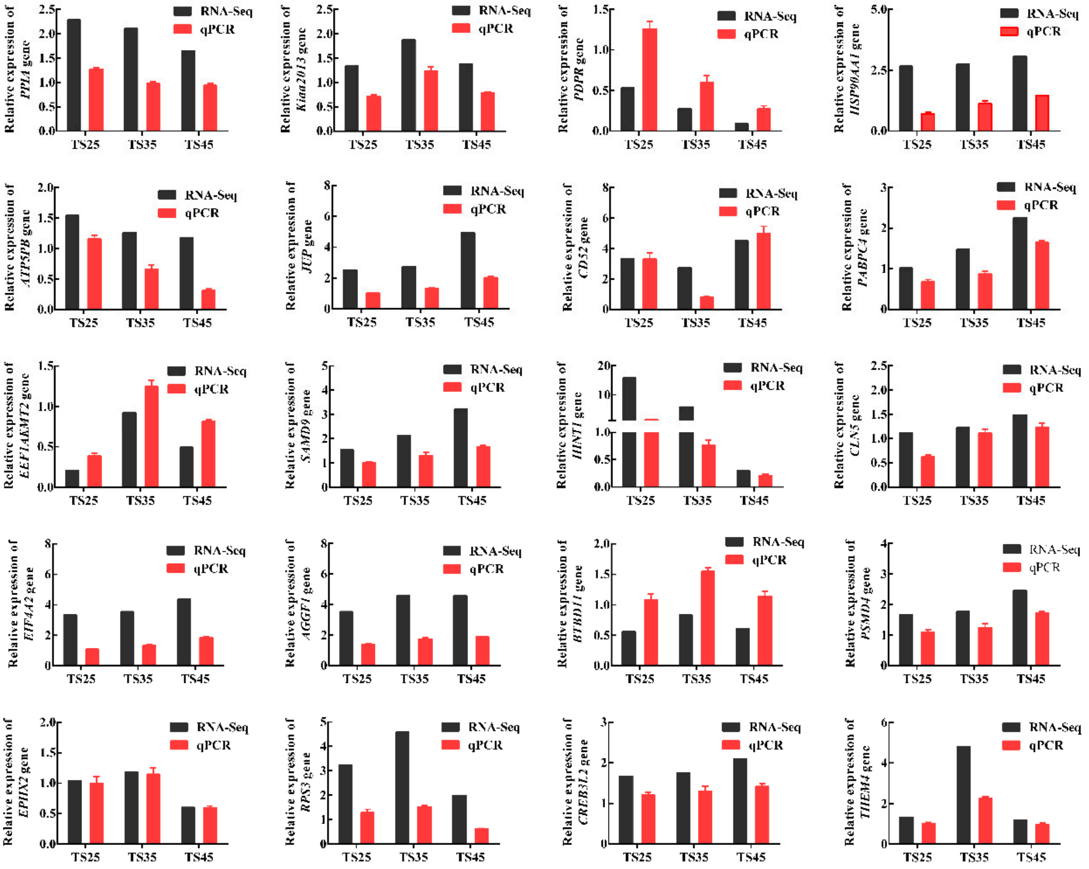

Figure S2. Verification results of twenty DEmRNAs by qPCR. QPCR date shown on graph are means ± SEM. (**TS25**) 2,500 m altitude Tibetan sheep. (**TS35**) 3,500 m altitude Tibetan sheep. (**TS45**) 4,500 m altitude Tibetan sheep.

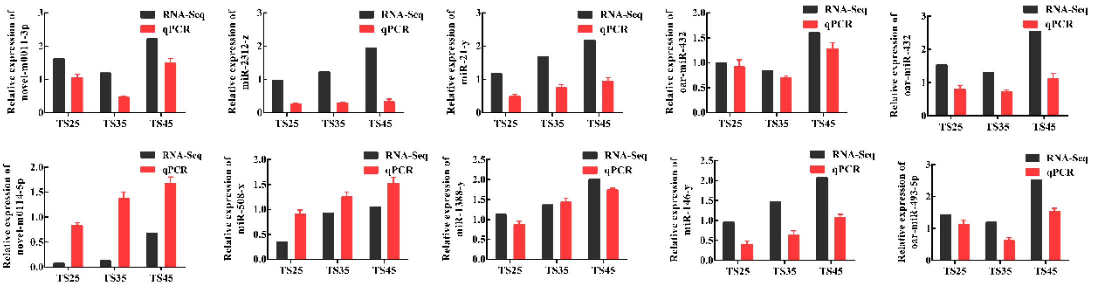

Figure S3. Verification results of ten DEmiRNAs by qPCR. QPCR date shown on graph are means ± SEM. (**TS25**) 2,500 m altitude Tibetan sheep. (**TS35**) 3,500 m altitude Tibetan sheep. (**TS45**) 4,500 m altitude Tibetan sheep.
