# Supplementary material for: Changes in the Mitochondrial Dynamics and Functions Together with the mRNA/miRNA Network in the Heart Tissue Contribute to Hypoxia Adaptation in Tibetan Sheep
Source: Animals (Basel). 2022 Feb 25;12(5):583. doi: 10.3390/ani12050583 (PMC8909807; doi:10.3390/ani12050583)
Supplement: Supplementary file 1 [file animals-12-00583-s001.zip › Supplementary material 1/Supplementary Table.pdf]

Supplementary Tables:

Table S1. The specific primer information of DEmRNAs were verified by qPCR.

Gene	GenBank ID	Primer sequence (5′→3′)	Product size (bp)	Annealing temperature/℃	Application
PP1A	NM_001308578.1	F: TGGCAAGTCCATCTATGGCG<br>R: ATGCTTGCCATCCAACCACT	157	60	qPCR
KIAA2013	XM_042257240.1	F: CTCTTCAGTCCAGGTGTGGAA<br>R: ATCTGGTCCCGCTCCCTATG	149	60	
PDPR	XM_027977618.2	F: GTAAAGGGAGGCTCTGTGTGAA<br>R: GCATCGCGTCTGTCAGCAAC	84	60	
HSP90AA1	XM_027957416.2	F: TTCAAACTCGTCAGACGCCC<br>R: TGGTGAGGGTTCGATCTTGC	125	60	
ATP5PB	XM_004002318.4	F: TACCTGACAATGCTGTCCCG<br>R: TTGCCTGCAATACCCCTGG	100	60	
JUP	XM_027974516.2	F: CTCCAAACGCAGCCACCAT<br>R: CGAGTGGATGCCAGAGTCAT	101	60	
CD52	NM_001368857.1	F: ATCTCCAAAAGTGCTGCCCA<br>R: CCAGGACTCCAGTTTGTATCTGA	117	60	
PABPC4	XM_042246030.1	F: AGCTGCTCAGAAGGATTCAAA<br>R: TGGTTCTTGGTGTTGAGGTCC	102	60	
EEF1AKMT2	XM_015103491.3	F: ACAGGAGAAATCTGGTTTGGAG<br>R: CCAAATTTTGCAAGTTCAACCAGC	140	60	
SAMD9	XM_042248377.1	F: AGGAGACAAGTAAACATCACAAC<br>R: GGGCATGAAGGAAATAACACAGG	180	60	
HINT1	XM_012178158.3	F: TGAGGATGACCAGTGTCTTGC<br>R: TTCTTCAGGCCCAGATCAGC	177	60	
CLN5	NM_001082595.1	F: AGTAGCAACCATATCAGGAGGC<br>R: CTTTTTCGGGCTGGCTTGGA	112	60	
EIF4A2	XM_027957453.2	F: GCTGTCTTTTCAGTCCGGCA<br>R: GGCCGCCATGTTCTCTGTTA	82	60	
AGGF1	XM_012180676.4	F: CCTCGAATTCACAGGAGCCA<br>R: TCTGCTGCAGCTCTCAAACT	94	60	
BTBD1	XM_042242645.1	F: TGACCGGATCAGGTTCACAG<br>R: CCGTGGGGCCATGAATAGAG	80	60	
PSMD4	NM_001104928.1	F: TGGTCTAGGAGAACCGGTCG<br>R: CACTGTTGTCCACGCAAACC	81	60	
EPHX2	XM_042243107.1	F: GCCGTGGACATGAAAGGCTA<br>R: CCTGAGCGATGCCCAACTTA	118	60	
RPS3	XM_004016328.5	F: CCCTAAGAAGCCTCTGCCTG<br>R: GCGGCCAAGGAGACCTATTA	153	60	
CREB3L2	XM_027968851.2	F: GACTTGTCCCCTCTGTCACG<br>R: GGCATGAGGAATCAACCCCA	102	60	
THEM4	XM_027975113.2	F: GACGGCCTGGGCTTTGAATA<br>R: CACCTCCATGAAGGAATCCAGG	115	60	
RPL19	XM_012186026.3	F: AATGCCAATGCCAACTC<br>R: CCCTTTCGCTACCTATACC	151	60	Reference gene
β-actin	NM_001009784.3	F: GCTGTATTCCCCTCCATCGT<br>R: GGATACCTCTCTTGCTCTGG	97	60	

Table S2. The specific primer information of DEmRNAs were verified by qPCR.

miRNA	Forward primer sequence (5′→3′)	Reverse primer sequence (5′→3′)	Application
miR-1388-y	TAATCTCAGGTTCGTCAGCCCG	mRQ 3′ primer (the same below)	qPCR
miR-508-x	CGCTACTCCAGAGGGTGTCATTC		
miR-146-y	TGCCCTAGGGACTCAGTTCTG		
novel-m0114-5p	TCATGGACAAGCTGTGTGGCA		
miR-432-x	CGTCTTGGAGTAGGTCATTGGGAA		
oar-miR-493-5p	CGCGTTGTACATGGTAGGCTTTCATT		
oar-miR-432	CTCTTGGAGTAGGTCATTGGGTGG		
novel-m0011-3p	CGCAAAGCCCAAACGAACTTTTTGT		
miR-21-y	CAACAGCAGTCGATGGGCTG		
miR-2312-z	CGCGAAAACCTGAATGAACTTATCGG		
U6	ACGGACAGGATTGACAGATT	TCGCTCCACCAACTAAGAA	Reference gene
18S	GTGGTGTTGAGGAAAGCAGACA	TGATCACACGTTCCACCTCATC	

Table S3. Overview of the reads and quality control of the 12 libraries of the mRNA sequencing from heart tissue of Tibetan sheep.

Samples	Raw dates	Clean date (bp)	Clean reads	Q20 (%)	Q30 (%)	GC (%)	Total mapped (%)	Unique mapped (%)
TS25-1	52,438,044	7,722,757,719	51,832,014	7,569,587,009 (98.02%)	7,278,354,660 (94.25%)	3,778,936,844 (48.93%)	50,022,319 (96.79%)	45,569,911 (88.17%)
TS25-2	40,532,686	5,961,146,045	39,913,902	5,843,395,824 (98.02%)	5,605,990,742 (94.04%)	2,914,627,392 (48.89%)	38,839,248 (97.54%)	35,698,373 (89.65%)
TS25-3	37,641,638	5,518,848,781	36,951,444	5,408,095,609 (97.99%)	5,184,161,006 (93.94%)	2,679,017,708 (48.54%)	35,896,587 (97.41%)	32,883,365 (89.23%)
TS25-4	36,673,766	5,409,888,006	36,211,232	5,308,023,556 (98.12%)	5,098,460,498 (94.24%)	2,626,025,203 (48.54%)	35,239,534 (97.55%)	32,291,835 (89.39%)
TS35-1	51,230,844	7,559,983,984	50,702,270	7,409,134,814 (98.00%)	7,122,152,279 (94.21%)	3,681,900,499 (48.70%)	48,857,374 (96.60%)	44,624,922 (88.24%)
TS35-2	36,486,182	5,350,596,612	35,848,724	5,245,158,103 (98.03%)	5,031,535,650 (94.04%)	2,556,687,013 (47.78%)	34,752,193 (97.22%)	31,706,201 (88.70%)
TS35-3	38,087,166	5,601,473,419	37,487,256	5,488,801,101 (97.99%)	5,259,521,340 (93.90%)	2,682,399,494 (47.89%)	36,355,096 (97.25%)	33,167,695 (88.72%)
TS35-4	41,890,282	6,161,110,959	41,253,662	6,037,817,325 (98.00%)	5,787,657,704 (93.94%)	2,957,693,620 (48.01%)	40,037,341 (97.34%)	36,566,526 (88.90%)
TS45-1	57,047,798	8,410,349,118	56,406,366	8,249,638,545 (98.09%)	7,940,258,407 (94.41%)	4,151,544,963 (49.36%)	54,398,619 (96.92%)	50,010,008 (89.10%)
TS45-2	38,744,938	5,703,075,667	38,171,996	5,582,064,063 (97.88%)	5,343,544,765 (93.70%)	2,737,300,929 (48.00%)	36,981,929 (97.32%)	33,971,358 (89.40%)
TS45-3	39,797,284	5,859,859,462	39,235,458	5,741,010,565 (97.97%)	5,502,908,424 (93.91%)	2,848,130,715 (48.60%)	38,026,361 (97.44%)	34,999,744 (89.68%)
TS45-4	39,436,788	5,789,924,579	38,772,316	5,664,699,657 (97.84%)	5,420,688,641 (93.62%)	2,828,894,666 (48.86%)	37,608,406 (97.35%)	34,538,207 (89.40%)

Table S4. Overview of the reads and quality control of the 12 libraries of the miRNA sequencing from heart tissue of Tibetan sheep.

Samples	Raw reads	High quality reads	Clean tags	Match genome (%)	Not match exon (%)
TS25-1	8,998,259 (100%)	8,918,227 (99.1106%)	8,894,072 (99.7292%)	6,775,008 (76.17%)	8,863,799 (99.66%)
TS25-2	15,836,840 (100%)	15,714,524 (99.2276%)	15,650,919 (99.5952%)	11,951,266 (76.36%)	15,557,978 (99.41%)
TS25-3	12,863,043 (100%)	12,772,606 (99.2969%)	12,720,617 (99.5930%)	9,561,405 (75.16%)	12,619,315 (99.20%)
TS25-4	12,645,103 (100%)	12,556,923 (99.3027%)	12,503,917 (99.5779%)	9,410,228 (75.26%)	12,450,586 (99.57%)
TS35-1	11,580,446 (100%)	11,468,520 (99.0335%)	11,433,349 (99.6933%)	8,834,054 (77.27%)	11,412,562 (99.82%)
TS35-2	15,128,103 (100%)	15,021,639 (99.2963%)	14,954,031 (99.5499%)	11,281,087 (75.44%)	14,844,695 (99.27%)
TS35-3	12,708,893 (100%)	12,621,979 (99.3161%)	12,570,388 (99.5913%)	9,501,310 (75.58%)	12,492,083 (99.38%)
TS35-4	12,785,289 (100%)	12,646,746 (98.9164%)	12,577,249 (99.4505%)	9,462,170 (75.23%)	12,495,343 (99.35%)
TS45-1	9,308,060 (100%)	9,243,247 (99.3037%)	9,223,364 (99.7849%)	7,314,664 (79.31%)	9,199,355 (99.74%)
TS45-2	13,555,629 (100%)	13,443,656 (99.1740%)	13,369,350 (99.4473%)	10,078,220 (75.38%)	13,233,318 (98.98%)
TS45-3	10,447,470 (100%)	10,370,054 (99.2590%)	10,334,989 (99.6619%)	7,988,228 (77.29%)	10,275,477 (99.42%)
TS45-4	14,437,359 (100%)	14,312,563 (99.1356%)	14,257,869 (99.6179%)	10,872,250 (76.25%)	14,141,986 (99.19%)
